# Multidrug-Resistant Tuberculosis Treatment in North Korea: Is Scale-Up Possible?

**DOI:** 10.1371/journal.pmed.1002062

**Published:** 2016-08-02

**Authors:** Kwonjune J. Seung, Molly Franke, Stephen W. Linton

**Affiliations:** 1 Eugene Bell Foundation, New Freedom, Pennsylvania, United States of America; 2 Partners In Health, Boston, Massachusetts, United States of America; 3 Brigham and Women's Hospital, Division of Global Health Equity, Boston, Massachusetts, United States of America; 4 Harvard Medical School, Boston, Massachusetts, United States of America

## Abstract

Kwonjune Seung and colleagues describe the Eugene Bell Foundation's experience of treating MDR-TB in North Korea.

**Summary Points**

- Multidrug-resistant tuberculosis (MDR TB) is one of the most serious public health problems facing North Korea, which has been included as one of the 30 countries on the World Health Organization's (WHO) high-burden MDR TB country list.
- Current international funding for TB control in North Korea is dominated by the Global Fund for AIDS, Tuberculosis and Malaria (GFATM), which focuses almost exclusively on drug-susceptible TB.
- The Eugene Bell Foundation (EBF) began treating MDR TB patients in collaboration with the North Korea Ministry of Public Health (MOPH) in 2008. Since then, close to 4,000 patients have been enrolled in treatment, over 1,000 patients in 2015 alone.
- The North Korean MDR TB treatment program has demonstrated better results than many other countries. MDR TB treatment should be scaled up immediately to the many North Koreans who need it.

## Multidrug-Resistant Tuberculosis in North Korea

Tuberculosis (TB) is one of the most serious public health problems facing the Democratic People's Republic of Korea (DPRK; North Korea), with an estimated incidence of 442/100,000 population [1]. Until recently, however, drug-resistant TB was not considered a major problem in DPRK, even though high rates have been documented in neighboring countries such as Russia and China. There has never been an epidemiological survey of drug-resistant TB in North Korea, but laboratory testing of strains collected for clinical purposes suggests that drug-resistant strains may indeed be widespread [2].

Even moderate rates of drug resistance, particularly multidrug resistance (MDR), defined as *Mycobacterium tuberculosis* resistant to isoniazid and rifampicin, would have serious ramifications for TB control efforts in North Korea. MDR TB requires 18–24 months of treatment with weak second-line TB drugs that have a wide variety of noxious side effects, and it is difficult to treat even in specialized centers in resource-rich settings [3,4].

The North Korean government has actively sought international partnerships in the area of TB control. In 2003, it received a donation of quality-assured first-line TB drugs from the Global Drug Facility, an initiative of the Stop TB Partnership in Geneva. In 2010, the Global Fund to Fight AIDS, Tuberculosis and Malaria (GFATM) started a major project to strengthen TB control in North Korea. The United Nations Children's Fund (UNICEF), the principal recipient, and the World Health Organization (WHO), the technical lead, are responsible for implementation of this project. Over US$52 million has been disbursed, but the project has almost exclusively focused on drug-susceptible TB [5].

The DPRK Ministry of Public Health (MOPH) first began diagnosing and treating MDR TB in 2008 with support from the Eugene Bell Foundation (EBF), a United States- and South Korea-based nongovernmental organization. The number of patients treated within this program has grown considerably, but the EBF program does not cover the entire country. In addition to the second-line TB drugs procured by EBF, the MOPH is able to procure a smaller quantity of second-line TB drugs via GFATM funds, but the total quantity continues to be inadequate to meet the need.

Even with the limited epidemiological data available, it is clear that significantly more second-line TB drugs would be needed to provide access to treatment to all MDR TB patients in North Korea. Yet it is already challenging for North Korea to deliver first-line TB drugs to over 100,000 patients annually. Even if adequate funding were available, it is reasonable to ask whether the MOPH would be able to scale up treatment for MDR TB throughout the country. In this article, we present outcomes of patients enrolled in the EBF program in 2012 and discuss the implications for scale-up of MDR TB treatment in North Korea.

## Eugene Bell Foundation MDR TB Treatment Program

### Treatment Centers

**Fig 1:**
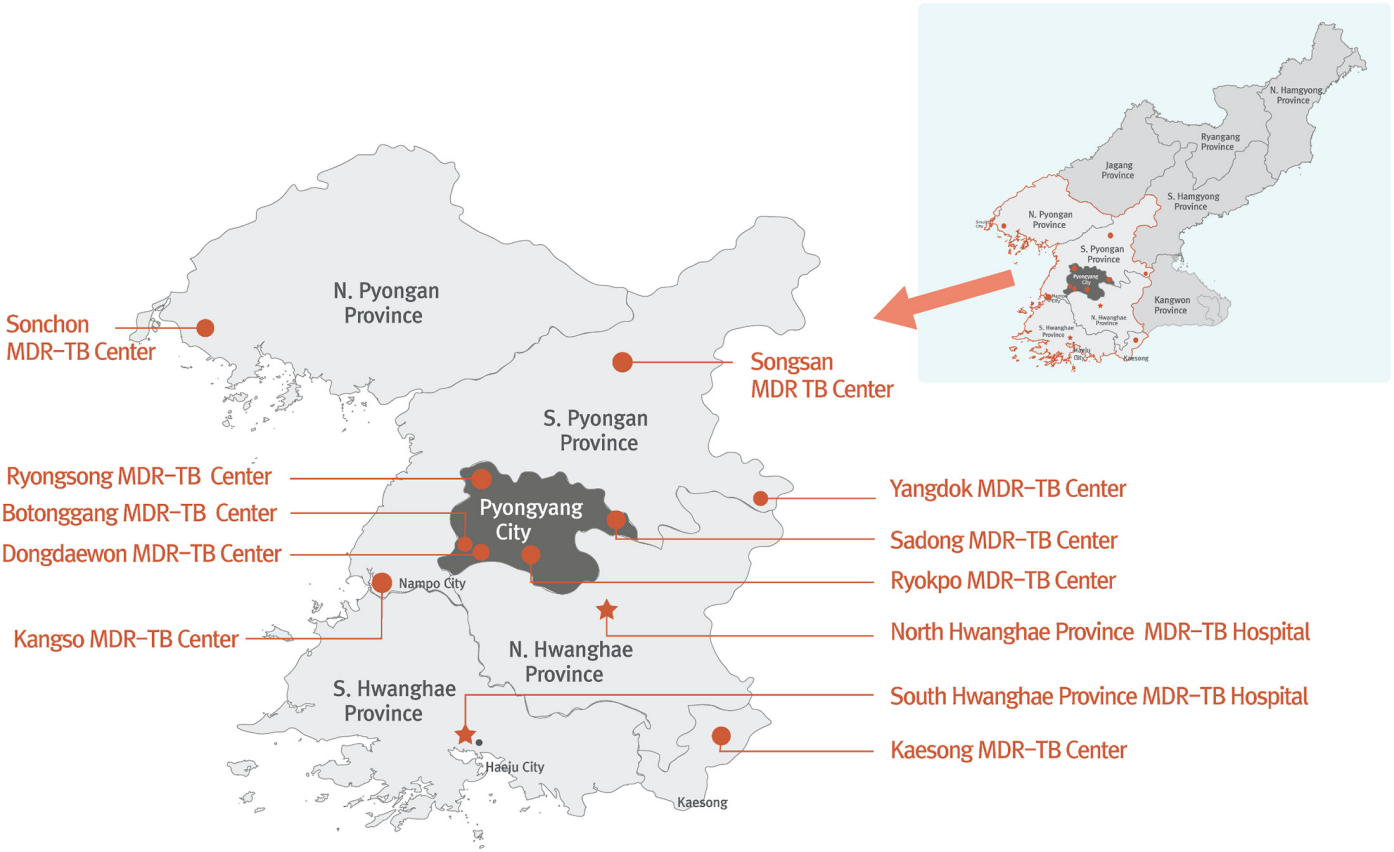
DPRK MDR TB treatment centers supported by the Eugene Bell Foundation (2015).

EBF support for MDR TB diagnosis and treatment is fully integrated into the extensive system of TB sanatoria that spans North Korea [2]. The number of sanatoria that have been designated as MDR TB treatment centers has grown steadily since 2008. Currently, EBF supports 12 MDR TB treatment centers in North and South Pyongan Provinces, North and South Hwanghae Provinces, and the cities of Pyongyang and Nampo (Fig 1). EBF visits each center once every six months to deliver new supplies of second-line TB drugs and laboratory consumables, enroll new patients, and monitor existing patients.

### Diagnosis

When the EBF program began in 2008, work was just starting on a national TB reference laboratory in Pyongyang. Since there was no way to do drug susceptibility testing (DST) in the country, an alternative system was developed for diagnosis and enrollment of patients that continues to this day. Before each EBF visit, potential candidates for MDR TB treatment are selected from those who have shown a lack of clinical or bacteriological response to standard directly observed treatment, short course (DOTS) regimens (Category I or II). These candidates are evaluated by provincial health facilities and eventually by the provincial TB referral center, which instructs candidates to present themselves to their designated MDR TB treatment site at the time of the EBF delegation visit. During the visit, EBF collects sputum samples from candidates under direct observation. In 2012, EBF began organizing a mobile GeneXpert laboratory for testing of these sputum samples with Xpert MTB/RIF on site. Patients with a positive Xpert MTB/RIF for rifampicin resistance are immediately enrolled in MDR TB treatment, and a second sputum sample is sent to the Korean Institute of Tuberculosis (KIT) for culture and first- and second-line DST. KIT is a South Korean laboratory that is part of the supranational reference laboratory network that monitors drug-resistant TB globally. Patients are generally required to have laboratory-confirmed rifampicin resistance in order to be enrolled in the EBF program, but exceptions are made for critically ill patients in the absence of DST results. Such patients were excluded from the outcomes presented here.

### Treatment

Since second-line DST results are generally delayed by six months from the time patients are enrolled in treatment, EBF provides a standardized MDR TB regimen to patients. In 2012, the standardized regimen included pyrazinamide, kanamycin, levofloxacin, prothionamide, cycloserine, and para-aminosalicylic acid (PAS), consistent with WHO guidelines [6,7]. The duration of treatment generally lasts 18–24 months, depending on the timing of smear and culture conversion. Patients are admitted to the sanatorium to begin treatment, and many live at the sanatorium throughout the full course of treatment. Patients can be discharged for outpatient treatment if stable; such decisions are the responsibility of the treating doctor.

Response to treatment is monitored by sputum culture. Sputum samples are collected under direct observation during the EBF visits, treated with cetyl-pyridinium chloride (CPC) [8] on site, and then transported to KIT for culture and first- and second-line DST. Since 2014, monitoring cultures have also been done between EBF visits at the national TB reference laboratory in Pyongyang, with the aim of a quarterly culture monitoring schedule for all MDR TB patients. Finally, each sanatorium is responsible for monitoring MDR TB patients monthly with light-emitting diode (LED) fluorescent smear microscopy, with equipment and reagents supplied by EBF.

## Outcomes of MDR TB Treatment in North Korea

We analyzed the outcomes of all patients enrolled in treatment for MDR TB at all seven (at that time) EBF-supported sanatoria between January 1 and December 31, 2012. During the study period, 353 patients with documented rifampicin resistance were enrolled in treatment. Baseline characteristics of these patients are shown in Table 1. Of these, 283 had full first- and second-line DST from KIT (Table 2); 70 were diagnosed only with Xpert MTB/RIF. Two hundred and fifty patients (70.8%) were successfully treated, which was defined as cured (230 patients) or completed (20 patients) classification. Sixty-two patients (17.6%) were classified as treatment failures, and 36 patients (10.2%) died during treatment. Five patients (1.4%) were lost to follow-up.

**Table 1:** Baseline characteristics of patients with MDR TB in North Korean treatment sites supported by EBF (*n* = 353).

Characteristic	*n* (%)	Median [interquartile range]
Male	223 (63.2)	
Age		39.3 [31.2–45.9]
Resistance category		
MDR but not pre-XDR^†^ or XDR^‡^	210 (59.5)	
Pre-XDR	54 (15.3)	
XDR	19 (5.4)	
Unknown (no second-line DST)	70 (19.8)	
Chest radiograph findings (*n* = 290)		
Bilateral disease	239 (82.4)	
Cavitary disease	231 (79.7)	
Destroyed lung	56 (19.3)	
None of the above	76 (26.2)	
Number of previous courses of TB treatment (*n* = 313)		
≤1 previous course of treatment	15 (4.8)	
2 previous courses of treatment	194 (62.0)	
3 previous courses of treatment	70 (22.4)	
≥4 previous courses of treatment	34 (10.9)	
Body mass index (BMI) (kg/m^2^) (*n* = 318)		
Severely low BMI (<16 kg/m^2^)	40 (12.6)	
Low BMI (16 to 18.5 kg/m^2^)	110 (34.6)	
Normal (≥18.5 kg/m^2^)	168 (52.8)	

^†^ Pre-XDR: MDR plus resistance to a second-line injectable or a fluoroquinolone, but not both.

^‡^ XDR: MDR plus resistance to a second-line injectable and a fluoroquinolone.

**Table 2:** Baseline DST patterns of patients with MDR TB in North Korean treatment sites supported by EBF (*n* = 283).

Resistance pattern	*n* (%)
MDR without second-line drug resistance	194 (68.6)
Isoniazid and rifampicin	46 (16.3)
Isoniazid, rifampicin, and ethambutol	16 (5.7)
Isoniazid, rifampicin, and streptomycin	54 (19.1)
Isoniazid, rifampicin, ethambutol, and streptomycin	48 (17.0)
Isoniazid, rifampicin, ethambutol, streptomycin, and pyrazinamide	14 (5.0)
MDR with second-line drug resistance	89 (31.4)
Pre-XDR^†^	54 (19.1)
XDR^‡^	19 (6.7)

^†^ Pre-XDR: MDR plus resistance to a second-line injectable or a fluoroquinolone, but not both.

^‡^ XDR: MDR plus resistance to a second-line injectable and a fluoroquinolone.

These outcomes answer the most important concern about scaling up MDR TB treatment in North Korea: whether the health system can effectively deliver such a complicated therapeutic intervention. These patients were suffering from advanced disease, as evidenced by the high frequency of bilateral and cavitary disease on chest radiograph, low body mass index (BMI), and history of multiple courses of treatment with first-line TB drugs (Table 1). Even so, the treatment outcomes compare favorably with those from other countries, including South Korea. Globally, for all patients enrolled in MDR TB treatment in 2012, WHO reported that the proportion of patients who successfully completed treatment was only 50% [1]. Published MDR TB treatment success rates from South Korea have been similar to the global average. For example, in a large cohort of 1,407 MDR TB patients from a mix of public and private clinics and hospitals, Kim et al. reported a success rate of 45.3% [9]. Compared to this South Korean cohort, our North Korean cohort had a similar proportion with extensive drug resistance (XDR; defined as MDR plus resistance to second-line injectables and fluoroquinolones) and a higher rate of bilateral and cavitary disease on chest radiograph. In a cohort of 202 MDR TB patients from three South Korean public hospitals with a higher rate of second-line drug resistance than this North Korean cohort, Jeon et al. reported a success rate of 37.1% [10].

Success rates in these two South Korean cohorts cannot straightforwardly be compared with that of the EBF cohort, since the South Korean studies used a slightly different definition of cure. Furthermore, South Korean patients benefit from many MDR TB treatment options that would improve outcomes in North Korea, such as widespread access to laboratory diagnosis, earlier initiation of appropriate treatment, treatment regimens that are individualized according to DST, and the availability of many more second-line TB drugs, especially those effective against pre-XDR and XDR strains. Nevertheless, there was an impressively low lost to follow-up rate (1.4%) in the North Korean cohort; the two South Korean studies reported lost to follow-up rates of 32.2% and 37.1%, respectively.

High MDR TB treatment lost to follow-up rates are commonly reported in both resource-limited and resource-rich settings. In a large meta-analysis of 18,294 patients across 31 countries, Toczek et al. found a pooled proportion lost to follow-up rate of 14.8%. Compared to these studies, the 1.4% lost to follow-up rate in this North Korean cohort would have been among the lowest in the meta-analysis, which included studies that reported rates from 0.57% to 55.6% [11]. The very low lost to follow-up rate in North Korea is noteworthy because it was much higher in the early years of the program, when neither patients nor clinicians had any experience with second-line TB drugs. By 2012, after considerable efforts to educate patients and clinicians by the MOPH and EBF, the program had matured and lost to follow-up rates had dropped considerably.

Our analysis of covariables associated with poor outcome (S1 Text and S1 Table) suggest that scaling up MDR TB treatment would likely result in higher cure rates if patients could be diagnosed and started on effective treatment in a timely manner. The vast majority of these patients had highly extensive disease, such as bilateral and cavitary disease. Seventeen percent of patients had a destroyed lung, a manifestation of advanced TB. Almost all patients had received multiple courses of treatment with WHO Category I and II regimens, a dangerous practice that drives the development of highly resistant strains [12].

Increasing access to effective treatment would also address the most important risk factor for poor outcome—resistance to second-line drugs created by irregular or inadequate treatment outside of the MDR TB treatment program. The XDR proportion in this cohort was 6.7%, and the pre-XDR (defined as MDR plus resistance to second-line injectables or to fluoroquinolones, but not both) proportion was 19.1% (Table 2), both surprisingly high considering that none of the patients had ever been formally treated with second-line TB drugs. Since all patients were tested prior to treatment initiation, this suggests that some patients had taken second-line TB drugs informally, or had been infected by someone who did. Tight control of second-line drugs within the MOPH should not be assumed to completely restrict access to second-line drugs outside of it. In many other countries, MDR TB patients are often young and highly motivated and will drain all of their limited resources to obtain small amounts of second-line TB drugs in informal markets. Unfortunately, this type of treatment is generally irregular and ineffective, and often results in the creation of even more resistant strains that spread to family and neighbors. New TB drugs such as bedaquiline, delamanid, and linezolid can and should be introduced to improve treatment of highly resistant MDR strains, but such efforts will be futile if patients do not have access to MDR TB treatment.

## Scaling up MDR TB treatment in North Korea

**Fig 2:**
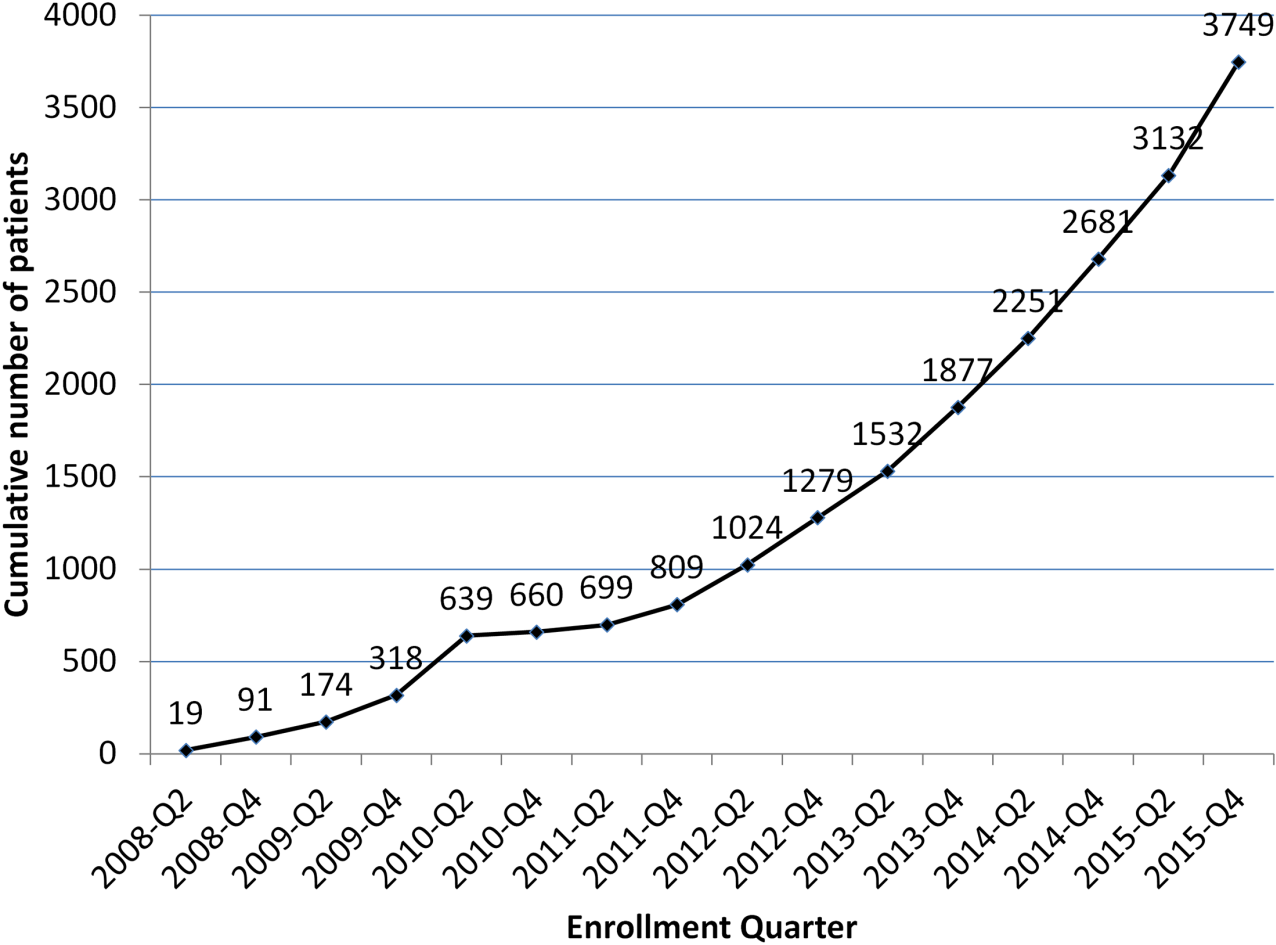
Cumulative Eugene Bell Foundation enrollment (2008–2015).

The encouraging patient outcomes demonstrated by EBF raise the question of why so little funding has been made available for MDR TB diagnosis and treatment in North Korea. The rate of patient enrollment has steadily increased, with close to 4,000 patients enrolled since 2008, and over 1,000 in 2015 alone (Fig 2). There is no doubt that thousands of lives have been saved, and even more MDR TB infections prevented. But from an epidemiological point of view, the program is just “feeding a biscuit to an elephant,” as the Koreans say. There are whole provinces with no access to the program, and only one or two treatment centers covering those provinces that are included.

Besides EBF, other sources of external funding have committed a surprisingly small amount for MDR TB diagnosis and treatment. Since 2010, the largest source of external funding for TB control in North Korea has been the GFATM. According to the 2015 GFATM Concept Note, the GFATM plans for 300 MDR TB patients to be treated in 2016, 400 in the second year, and 450 in the third year [13]. In a country where WHO estimates 3,800 new MDR TB patients annually, these numbers are epidemiologically meaningless [1].

These funding decisions are even more surprising as the cost of MDR TB diagnosis and treatment continues to become more affordable. According to current WHO guidelines, priority for rapid DST should be given to retreatment (Category II) patients, close contacts of drug-resistant TB patients who have been diagnosed with active TB, and patients not responding to first-line treatment (Category I regimens) [14]. For DPRK, this would require approximately 15,000 Xpert MTB/RIF cartridges per year, at a cost of less than US$150,000 annually [15]. EBF's increasing patient enrollment undermines the argument that a national survey of drug-resistant TB is required before scaling up MDR TB treatment. Minimal effort has been needed to diagnose thousands of patients with MDR TB, even in rural areas. In fact, the number of diagnosed patients routinely outstrips EBF's limited funds.

No matter the cost of scale-up, it is certainly dwarfed by the human cost of not intervening to stop this epidemic. MDR TB is an infectious airborne disease that will continue to spread in North Korea unless there is widespread access to effective treatment. North Korea has recently been included as one of the 30 countries on the WHO high-burden MDR TB country list, yet external funding focuses almost exclusively on drug-susceptible TB. The EBF program has demonstrated encouraging results that are better than many countries, including South Korea. MDR TB treatment should be scaled up immediately to the many North Koreans who need it.

## Supporting Information

S1 Table Univariate and multivariate associations between clinical characteristics and poor outcome (*n* = 348). (DOCX)

S1 Text Analysis of covariables associated with a poor outcome. (DOCX)

S2 Text Korean translation of the manuscript by Seemoon Choi and Hyemin Lee. (DOCX)

## Abbreviations

CPC: cetyl-pyridinium chloride
DOTS: directly observed treatment, short course
DPRK: Democratic People’s Republic of Korea
DST: drug susceptibility testing
EBF: Eugene Bell Foundation
GFATM: Global Fund for AIDS, Tuberculosis and Malaria
KIT: Korean Institute of Tuberculosis
LED: light-emitting diode
MDR: multidrug resistance
MOPH: North Korea Ministry of Public Health
PAS: para-aminosalicylic acid
TB: tuberculosis
UNICEF: United Nations Children’s Fund
WHO: World Health Organization
XDR: extensive drug resistance
