# Supplementary material for: Multidrug-Resistant Tuberculosis Treatment in North Korea: Is Scale-Up Possible?
Source: PLoS Med. 2016 Aug 2;13(8):e1002062. doi: 10.1371/journal.pmed.1002062 (PMC4970717; doi:10.1371/journal.pmed.1002062)
Supplement: S1 Table — (DOCX) [file pmed.1002062.s001.docx]

Supplemental Table: Univariate and multivariate associations between clinical characteristics and poor outcome (N=348)

| **Variable** | **N** | **Poor outcome n(%)*** | **Unadjusted OR** | **P-value** | **Adjusted OR****** | **P-value** |
| --- | --- | --- | --- | --- | --- | --- |
| Gender | 348 |  |  |  |  |  |
| Female | 128 | 35 (27.3) | Reference |  |  |  |
| Male | 220 | 63 (28.6) | 1.07 (0.66 – 1.73) | 0.80 |  |  |
| Median age (years) [IQR]** | 348 | 36.7 [30.0, 44.7] | 0.99 (0.97, 1.01) | 0.35 |  |  |
| Resistance category | 348 |  |  |  |  |  |
| Multidrug-resistance but not pre-extensive drug resistance or extensive drug resistance | 207 | 45 (21.7) | Reference |  | Reference |  |
| Extensive drug resistance | 19 | 8 (42.1) | 2.62 (0.99 – 6.90) | 0.05 | 2.22 (0.78 – 6.27) | 0.13 |
| Pre-extensive drug resistance | 52 | 24 (46.2) | 3.09 (1.63 – 5.84) | 0.0005 | 3.25 (1.66 – 6.37) | 0.0006 |
| Unknown (no second line DST) | 70 | 21 (30.0) | 1.54 (0.84 – 2.84) | 0.16 | 1.35 (0.71 – 2.58) | 0.36 |
| Chest radiograph findings | 286 |  |  |  |  |  |
| Did not have both bilateral and cavitary disease; no destroyed lung | 74 | 8 (10.8) | Reference |  | Reference |  |
| Bilateral and cavitary disease *** | 210 | 66 (31.4) | 2.83 (1.31, 6.14) | 0.008 | 2.72 (1.34 – 5.52) | 0.006 |
| Destroyed lung *** | 55 | 24 (43.6) | 2.16 (1.15 – 4.08) | 0.02 | 2.12 (1.01 – 4.43) | 0.05 |
| Previous TB treatments | 308 |  |  |  |  |  |
| < 2 | 205 | 59 (28.8) | Reference |  |  |  |
| >2 | 103 | 30 (29.1) | 1.02 (0.60 – 1.71) | 0.95 |  |  |
| BMI category | 313 |  |  |  |  |  |
| >18.5 (not underweight) | 165 | 37 (22.4) | Reference |  | Reference |  |
| 16 < BMI < 18.5 (underweight) | 108 | 32 (29.6) | 1.46 (0.84 – 2.53) | 0.18 | 0.97 (0.53 – 1.77) | 0.91 |
| BMI < 16 (severely or very severely underweight) | 40 | 14 (35.0) | 1.86 (0.88 – 3.93) | 0.10 | 1.06 (0.47 – 2.41) | 0.89 |

* Unless otherwise noted

** IQR: Interquartile range

*** Bilateral and cavitary disease and destroyed lung were not mutually exclusive categories

**** The final multivariable model included resistance category, chest radiograph findings, and BMI category
