# Supplementary material for: Multidrug-Resistant Tuberculosis Treatment in North Korea: Is Scale-Up Possible?
Source: PLoS Med. 2016 Aug 2;13(8):e1002062. doi: 10.1371/journal.pmed.1002062 (PMC4970717; doi:10.1371/journal.pmed.1002062)
Supplement: S1 Text — (DOCX) [file pmed.1002062.s002.docx]

# Supplemental text: Analysis of covariables associated with a poor outcome

# Methods

## Laboratory

At KIT, the TB Ag MPT64 Rapid kit (SD Bioline, South Korea), an immunochromatographic test using mouse monoclonal antibodies to detect the MPT64 protein, was used on positive cultures for identification of *Mycobacterium tuberculosis* complex. Culture and susceptibility testing were performed on Löwenstein-Jensen media to first- and second-line drugs. Critical concentrations were as follows (mcg/ml): isoniazid 0.2, rifampicin 40.0, ethambutol 2.0, streptomycin 10.0, kanamycin 40.0, amikacin 40.0, capreomycin 40.0, ofloxacin 2.0, prothionamide 40.0, cycloserine 30.0, PAS 1.0. Pyrazinamide susceptibility was determined using the pyrazinamidase test.

## Data collection

During the course of treatment, sanatoria doctors recorded information about each patient on a standard WHO MDR TB treatment card, which was subsequently transferred to a standard WHO MDR TB treatment register. These were digitally photographed on each EBF delegation visit, and covariable data, including gender, birthdate, and number of previous courses of TB treatment, were later abstracted. EBF delegation staff measured weight and height of each patient on the date of enrollment. EBF delegation staff also retrieved digital radiographs that were taken of patients on the date of enrollment by a mobile radiography unit originally provided by EBF. Chest radiographs were later reviewed and coded (KJS) according to three binary variables: bilateral disease, cavitary disease and destroyed lung.

## Outcome definitions

Outcome definitions were based on the WHO *Companion handbook to the WHO guidelines for the programmatic management of drug-resistant tuberculosis* (WHO/HTM/TB/2014.11). The only difference was in the definition of treatment failure. According to WHO definitions, a lack of conversion or bacteriological reversion is based on two consecutive positive cultures taken at least 30 days apart. In this study, a single positive culture in the last 12 months of treatment was considered evidence of reversion or lack of conversion. A bad outcome was defined as treatment failure or death.

## Statistical analyses

Data were described using counts and percentages for binary and categorical data and medians and interquartile ranges for continuous data. Univariable logistic regression analyses were performed to identify potential predictors of a poor outcome. Only baseline clinical and demographic variables with biological or social plausibility were analyzed. Those variables that predicted the outcome at a P value of ≤0.20 in univariable analyses were included in the multivariable model. We selected a threshold of ≤0.20 in order to retain in the final model statistically significant risk factors (i.e. those associated with treatment outcome at a P value <0.05) as well as factors that were not statistically significantly associated with treatment outcome but could nonetheless confound the relationship between other risk factors and treatment outcome. To account for missing covariable data, we performed multivariable analyses on data sets multiply imputed (N=5) using covariable and outcome data. Imputation was conducted using the Markov Chain Monte Carlo methods (SAS MI Procedure), and effect estimates were pooled across data sets. A p-value <0.05 was used to determine statistical significance.

# Analysis of covariables associated with a poor outcome

In univariable analyses, baseline infection with a pre-XDR or XDR strain was associated with higher odds of a poor outcome, as were radiographic findings of a destroyed lung or bilateral disease and cavitary disease (Table 3). These two factors, along with body mass index (BMI), were included in the final multivariable model. In a multivariable analysis, baseline infection with a pre-XDR strain was associated with three times the odds of a poor outcome, relative to infection with a MDR strain that was not pre-XDR or XDR (OR: 3.25, 95% CI: 1.66 – 6.37). Individuals with a baseline chest radiograph demonstrating bilateral and cavitary disease or those with a destroyed lung each had twice the odds of a poor outcome relative to those without this extensive lung damage (OR: 2.72, 95% CI: 1.34 – 5.52; OR 2.12, 95% CI: 1.01 – 4.43, respectively, Table 3). Although baseline infection with an XDR strain was associated with twice the odds of a poor outcome, this association was not statistically significant (OR: 2.22, 95% CI: 0.78 – 6.27).
