# Supplementary material for: Multidrug-Resistant Tuberculosis Treatment in North Korea: Is Scale-Up Possible?
Source: PLoS Med. 2016 Aug 2;13(8):e1002062. doi: 10.1371/journal.pmed.1002062 (PMC4970717; doi:10.1371/journal.pmed.1002062)
Supplement: S2 Text — (DOCX) [file pmed.1002062.s003.docx]

**제목:** 북한에서의 다제내성결핵 치료: 사업의 확대는 가능한가?

**저자:** Kwonjune J. Seung(승권준)^1,2,3,4^(교신 저자), Molly Franke^2,4^, Stephen W. Linton(인세반)^1^

**소속기관:**

1. Eugene Bell Foundation, P.O. Box 577, New Freedom, PA, USA

2. Partners In Health, 888 Commonwealth Ave., Boston USA

3. Brigham and Women's Hospital, Division of Global Health Equity, 75 Francis St., Boston USA

4. Harvard Medical School, 250 Longwood Ave., Boston, USA

**요약:**

- 다제내성결핵은 북한이 직면하고 있는 심각한 공공보건 문제다. 북한은 세계보건기구(World Health Organization)가 지정한 다제내성결핵으로 인한 질병부담이 높은 30개국에 속한다.
- 현재 북한의 결핵 퇴치를 위한 국제적인 지원은 주로 에이즈/결핵/말라리아 퇴치를 위한 세계기금(Global Fund Against AIDS, TB and Malaria)에서 주도적으로 진행하고 있는데, 대부분 약제감수성이 있는 일반 결핵에만 집중하고 있다.
- 유진벨 재단은 지난 2008년, 북한 보건성과의 협력 하에 다제내성결핵 환자 치료를 시작했다. 이후 약 4,000명의 환자들이 등록되어 치료를 받았고, 2015년 한 해에만 1,000여 명의 환자들이 새로 등록했다.
- 북한 다제내성결핵 치료 프로그램은 세계의 다른 국가들보다 치료 성적이 우수하다. 다제내성결핵 치료 프로그램이 한시라도 빨리 확대되어 더 많은 북한 환자들이 치료 혜택을 받아야 한다.

**북한 내 다제내성결핵**

결핵은 조선민주주의인민공화국(이하 북한)이 직면하고 있는 가장 심각한 공공보건 문제로, 인구 10만 명 당 442명의 새로운 결핵환자가 발생하고 있다[1]. 그러나 인접 국가인 러시아와 중국에서 최근까지도 내성결핵 발생률을 보이고 있음에도 불구하고, 약제내성결핵은 북한에서 중요한 문제로 인식되지 않았다. 아직까지 북한 내 내성결핵에 대한 역학조사가 이루어진 적은 없었으나, 임상 목적으로 수집된 결핵균주의 검사결과는 약제내성결핵이 북한에서 이미 널리 퍼져있다는 것을 시사한다[2].

특히 이소니아지드와 리팜피신에 내성에 있는 결핵균으로 정의되는 다제내성결핵을 비롯한 약제내성률이 어느 정도 수준만 되어도, 이는 북한 내 결핵 관리에 심각한 영향을 미친다. 다제내성결핵은 18-24개월의 치료기간이 필요하고, 다양한 종류의 부작용을 일으키는 약한 이차 항결핵제로 치료하며, 필요한 자원이 충분한 결핵 전문 센터에서도 치료하기가 매우 어렵다[3, 4].

북한 정부는 결핵 퇴치를 위한 국제적인 협력 관계를 적극적으로 모색해왔다. 지난 2003년, 제네바 Stop TB Partnership 프로그램인 Global Drug Facility은 품질이 보장된 일차 항결핵제를 후원했다. 2010년에는 에이즈/결핵/말라리아 퇴치를 위한 세계기금(Global Fund Against AIDS, TB and Malaria; 이하 세계기금)에서 북한 결핵 퇴치 사업을 강화하기 위한 주요 사업들을 착수했다. 프로젝트의 책임 기관인 유엔아동기금(UNICEF)와 전문 기술을 담당하는 세계보건기구(World Health Organization)가 이 프로젝트의 집행을 담당하고 있다. 미화로 5,200만 달러 이상이 지출되었는데, 이 사업은 약제감수성이 있는 결핵에 집중되었다[5].

북한 보건성은 2008년 처음으로 미국과 남한에 기반을 둔 비정부기구인 유진벨 재단의 지원을 받아 다제내성결핵에 대한 진단과 치료를 시작했다. 유진벨 프로그램에 등록되어 치료를 받은 환자 수는 눈에 띄게 증가했지만, 사업을 북한 전역으로 확대하기엔 한계가 있었다. 보건성은 유진벨 재단과 세계기금에서 이차 항결핵제를 지원받았으나, 이를 다 합해도 모든 수요를 채우기엔 역부족이다.

이용가능한 역학조사자료가 한정적이지만, 북한의 모든 다제내성결핵 환자들이 치료를 받으려면 훨씬 더 많은 이차 항결핵제가 필요한 것은 분명하다. 그러나 현재 북한 정부로서는 매년 10만 명이 넘는 환자들에게 일차 항결핵제를 제공하는 것조차도 이미 어렵다. 만약 충분한 재정적 지원이 이뤄지더라도, 보건성이 다제내성결핵 치료를 북한 전역으로 확대할 수 있는가에 대해 의문을 제기할 수 있다. 이 글에서는 2012년 유진벨 프로그램에 등록된 환자들의 치료 결과를 제시하고, 북한 내 다제내성결핵 치료 프로그램의 확장이 의미하는 바가 무엇인지 논의하고자 한다.

**유진벨 재단의 다제내성결핵 치료 프로그램**

**치료소**

유진벨 재단의 다제내성결핵 진단 및 치료 프로그램은 북한 전역의 결핵전달체계에 완전히 통합되어 운영되고 있다[2]. 다제내성결핵 치료소로 지정된 요양소 수는 2008년부터 꾸준히 증가했다. 현재 유진벨 재단에서는 12개 다제내성결핵 요양소를 지원하고 있으며, 이 요앙소들은 평안남∙북도, 황해남∙북도와 평양 및 남포시에 위치한다(그림 1). 유진벨 재단은 6개월에 한 번씩 각각의 센터를 방문하여 이차 항결핵제와 검사실 소모품을 전달하고, 새로운 환자를 등록하며, 기존 등록 환자들의 치료 경과를 모니터링한다.

**그림 1: 유진벨 재단이 지원하는 북한 다제내성결핵 요양소(2015)**


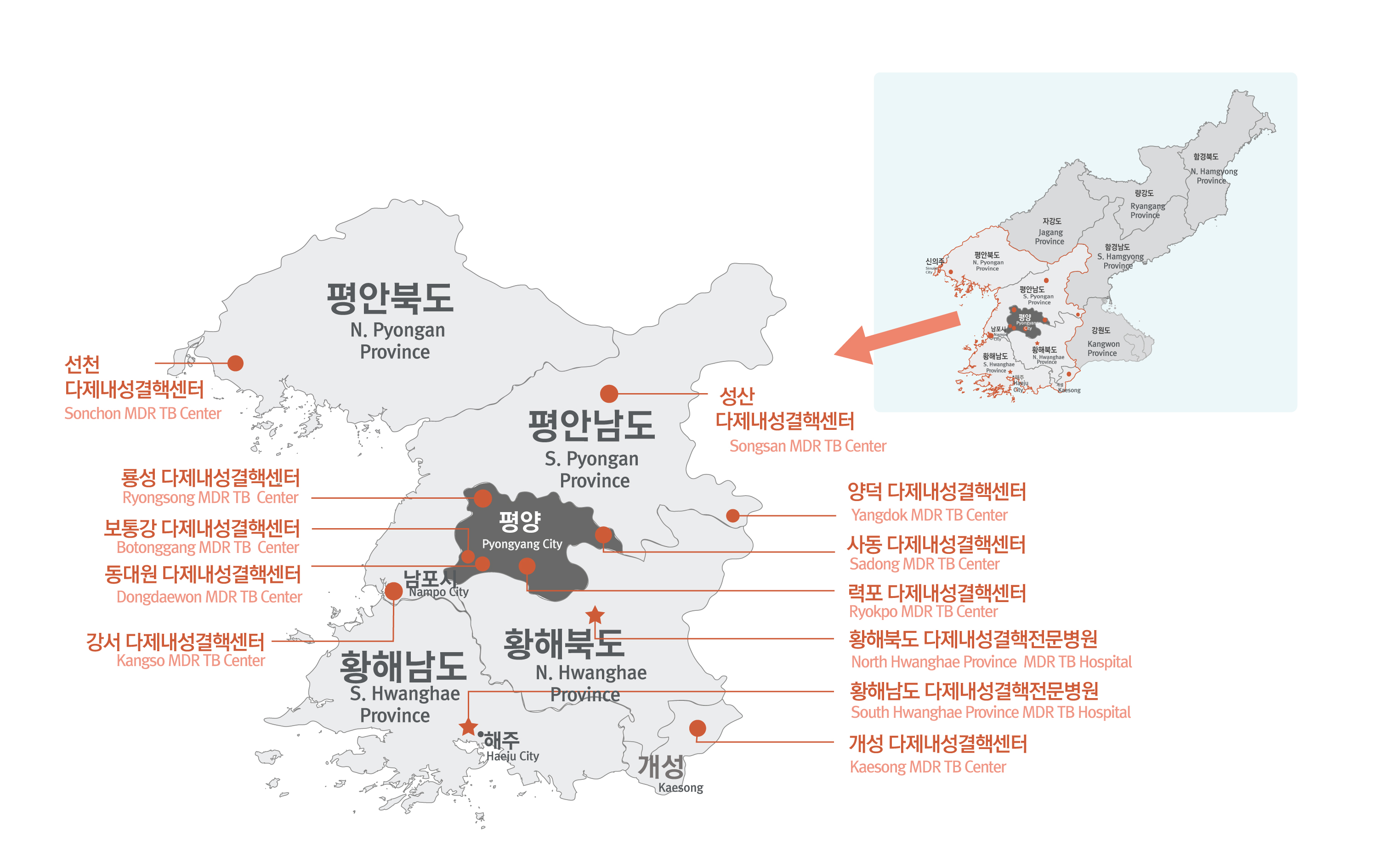


**진단**

2008년 유진벨 재단이 프로그램을 시작했을 때, 평양의 국가 결핵 표준 실험실도 업무를 막 개시했다. 북한 내에서는 약제감수성검사를 할 수 있는 방법이 없기 때문에, 유진벨 재단은 진단 및 환자 등록이 가능한 대안체계를 마련했는데, 지금까지도 그 체계 내에서 프로그램이 운영되고 있다. 유진벨 재단의 매 방북 전에, 표준 DOTS 처방(1부류 또는 2부류)에 임상적 세균학적 반응을 보이지 않은 사람들 중 다제내성결핵 치료 대상자가 선별된다. 이 대상자들은 도립 결핵병원(제3예방원)에서 검사를 받는다. 판별을 받은 대상자들은 유진벨 대표단이 방문하는 시점에 맞춰 지정된 요앙소에 찾아가도록 지시를 받는다. 방문 기간 동안, 이들 대상자들은 대표단의 직접 관찰 하에 객담을 채취한다. 2012년, 유진벨 재단은 현장에서 Xpert^®^ MTB/RIF로 객담을 바로 검사할 수 있는 기기를 도입했다. 리팜피신 내성을 판별하는 환자들은 현장에서 바로 유진벨 프로그램에 등록되며, 또 다른 객담 샘플은 남한의 결핵연구원으로 옮겨져서 배양 및 일차, 이차 항결핵제 약제감수성 검사를 실시한다. 결핵연구원은 국제 표준 실험 네트워크에 소속되어 있다. 환자들이 유진벨 프로그램에 등록되기 위해서는 일반적으로 검사로 확진된 리팜피신 내성이 있어야 하지만, 임상 상태가 위중한 환자들에겐 예외가 적용되기도 한다. 이런 환자들의 치료 결과는 분석에 포함되지 않았다.

**치료**

이차 항결핵제 약제감수성 결과는 일반적으로 환자가 치료 프로그램에 등록한 시점으로부터 6개월이 지난 후 확인할 수 있기 때문에, 유진벨 재단은 표준화된 다제내성결핵 처방을 환자들에게 제공한다. 2012년 표준화된 처방에는 세계보건기구에서 지침에 따라 pyrazinamide, kanamycin, levofloxacin, prothionamide, cycloserine, para-aminosalicylic acid (PAS)이 포함된다[6, 7]. 치료 기간은 일반적으로 18-24개월 정도이며 도말이나 배양 전환 시기에 따라 다를 수 있다. 치료를 받기 위해 요양소에 입원하는 환자 대부분은 치료 기간 내내 요양소에서 거주한다. 환자 상태가 안정적인 경우에는 외래 치료를 받기 위해 퇴원할 수 있으며, 이 결정은 치료를 담당하는 의료진이 내린다.

치료에 대한 반응은 객담 배양을 통해 모니터링하고 있다. 객담 샘플은 유진벨 재단이 방문 중 직접적인 관찰 하에 수집하며, 현장에서 cetyl-pyridinium chloride (CPC) [8]로 처리되고, 배양 및 일차, 이차 항결핵제 약제감수성 검사를 목적으로 결핵연구원으로 옮겨진다. 2014년부터는 모든 다제내성결핵 환자들에 대한 분기별 배양 검사를 목적으로, 유진벨 대표단의 방문 중간에 배양 검사는 평양에 위치한 국가 결핵 표준 실험실에서도 이루어진다. 최종적으로, 각 요양소는 유진벨 재단에서 제공한 장비와 시약을 이용해 LED 형광 도말 현미경검사를 통해 다제내성결핵 환자들을 월별로 모니터링하고 있다.

**북한 다제내성결핵 치료 결과**

우리는 2012년 1월 1일부터 12월 31일까지 당시 기준으로 유진벨 재단이 지원하는 총 7개 요양소의 다제내성결핵 치료 프로그램에 등록되어 있는 모든 환자들의 치료 결과를 분석하였다. 이 기간 동안, 리팜피신에 내성이 있는 353명의 환자들이 치료 프로그램에 등록하였다. 환자들의 기본 특성은 표1에서 제시되어 있다. 이 중, 283명은 결핵연구원에서 일차 및 이차 약제감수성 검사를 모두 마쳤고(표2), 70명은 Xpert^®^ MTB/RIF로만 진단 받았다. 250명(70.8%)은 성공적으로 치료를 마쳐 완치로 판명되었거나(230명), 치료를 완료하였다(20명). 62명(17.6%)은 치료 실패로 판명되었고, 36명(10.2%)은 치료 도중 사망하였다. 5명(1.4%)은 치료를 중단하였다.

**표1: 유진벨 재단 치료 프로그램에 등록된 북한 다제내성결핵 환자들의 기본 특징(N=353))**

| ***특징*** | ***N (%)*** | ***중간값 [사분범위]*** |
| --- | --- | --- |
| 남성 | 223 (63.2) |  |
| 연령 |  | 39.3 [31.2 - 45.9] |
| 내성 정도 |  |  |
| MDR^†^(Pre-XDR^*^, XDR^**^은 제외) | 210 (59.5) |  |
| Pre-XDR | 54 (15.3) |  |
| XDR | 19 (5.4) |  |
| 알 수 없음 (2차 계열 DST 결과 없음) | 70 (19.8) |  |
| 흉부 방사선 결과 (N=290) |  |  |
| 양측성 병변 | 239 (82.4) |  |
| 공동 | 231 (79.7) |  |
| 파괴 폐 | 56 (19.3) |  |
| 위 세 증상에 모두 없음 | 76 (26.2) |  |
| 일반 결핵치료 경험 횟수 (N=313) |  |  |
| 1 회 이하 | 15 (4.8) |  |
| 2 회 | 194 (62.0) |  |
| 3 회 | 70 (22.4) |  |
| 4 회 이상 | 34 (10.9) |  |
| 체질량지수(BMI) (kg/m2) (N=318) |  |  |
| 중증 저체중 (<16 kg/m2) | 40 (12.6) |  |
| 저체중 (16 to 18.5 kg/m2) | 110 (34.6) |  |
| 정상 (≥18.5 kg/m2) | 168 (52.8) |  |

^†^ MDR: 이소니아지드와 리팜피신에 내성이 있음.

* Pre-XDR: MDR이면서 이차 항결핵제 주사액이나 플루오로퀴놀론 중 하나에 내성이 있음.

** XDR: MDR이면서 이차 항결핵제 주사액과 플루오로퀴놀론 모두에 내성이 있음.

**표2: 유진벨 재단으로부터 지원 받는 북한 치료소에 다제내성결핵으로 등록된 환자들의 치료 전약제감수성검사 결과(N=283)**

| ***내성 양상*** | ***N (%)*** |
| --- | --- |
| 이차 항결핵제에 내성이 없는 다제내성결핵 | 194 (68.6) |
| Isoniazid, rifampicin | 46 (16.3) |
| Isoniazid, rifampicin, ethambutol | 16 (5.7) |
| Isoniazid, rifampicin, streptomycin | 54 (19.1) |
| Isoniazid, rifampicin, ethambutol, streptomycin | 48 (17.0) |
| Isoniazid, rifampicin, ethambutol, streptomycin, pyrazinamide | 14 (5.0) |
| 이차 항결핵제에 내성이 있는 다제내성결핵 | 89 (31.4) |
| Pre-XDR^*^ | 54 (19.1) |
| 광범위내성결핵(XDR)^**^ | 19 (6.7) |

* Pre-XDR: MDR이면서 이차 항결핵제 주사액이나 플루오로퀴놀론 중 하나에 내성이 있음.

** 광범위내성결핵(XDR): MDR이면서 이차 항결핵제 주사액과 플루오로퀴놀론 모두에 내성이 있음.

이 결과는 북한 내 다제내성결핵 치료 확대에 있어 가장 중요한 이슈인, 북한의 보건의료체계가 이렇게 복잡한 치료서비스를 효과적으로 전달할 수 있는가에 대한 질문에 답을 제시한다. 흉부 방사선 결과에서 양측 및 공동 발병, 낮은 체질량지수(BMI), 다수의 일차 항결핵제 치료 경험 등과 같이 이 환자들은 악화된 결핵으로 고통 받고 있었다 (표1). 세계보건기구가 2012년 한 해 동안 다제내성결핵 치료를 받은 전 세계의 모든 환자들 중 성공적으로 치료를 마친 비율이 50%[1]에 불과하다고 보고한 점을 감안할 때, 이 치료 결과는 남한을 포함한 다른 국가들보다 우수하다. 발표된 남한의 다제내성결핵 치료 성공률도 세계 평균과 비슷했다. 예를 들면, 공공 및 민간의 병의원 구분 없이 1,407명의 다제내성결핵 환자 집단에서 치료 성공률이 45.3%[9]였다고 Kim 등은 발표했다. 남한의 환자들과 비교했을 때, 유진벨 재단에서 지원한 북한 환자들은 광범위 약제 내성(XDR)비율은 비슷한 수준이었고, 흉부 방사선 결과에서 보이는 양쪽 및 공동 발병률은 다소 높았다. 이 연구에 포함된 북한 환자들보다 이차 항결핵제에 내성을 지닌 비율이 더 높은, 남한 내 세 곳의 공립 병원에서 202명의 다제내성결핵 환자 집단에서는 치료 성공률이 37.1%였다고 Jeon 등이 발표했다[10].

위에 언급한 남한의 두 연구에서 사용한 치료의 정의가 약간 다르기 때문에, 이 연구의 치료성공률을 유진벨 재단의 환자 집단과 직접적으로 비교할 수 없다. 더구나, 남한의 환자들은 각종 검사에 대한 접근성이 용이하고, 적절한 치료를 조기에 받을 수 있으며, 약제감수성 검사에 따른 개인별 처방, 특히 Pre-XDR 또는 XDR 검체에 효과적인 다양한 이차 항결핵제의 접근성 등과 같은 다제내성결핵 치료에 필요한 많은 혜택을 받을 수 있다. 이런 혜택을 유진벨 재단이 지원하는 북한의 환자들이 받게 된다면, 북한의 치료 결과 또한 향상될 수 있을 것이다. 그럼에도 불구하고, 북한 환자들의 치료 중단률은 1.4%로 놀라운 결과를 보인 반면, 앞서 인용한 두 편의 연구에서 남한 환자들의 치료 중단률은 각각 32.2%와 37.1%로 보고되었다.

다제내성결핵 치료에서 높은 치료 중단률은 자원이 제한된 곳과 충분한 곳 모두에서 흔히 보고되곤 한다. 31개국 18,294명의 환자들을 대상으로 메타 분석한 결과, Toczek 등은 치료 중단률이 14.8%라는 결과를 얻었다. 이 연구 결과와 비교했을 때, 북한의 1.4%라는 치료 중단률은 최소 0.57%에서 최대 55.6%까지로 집계되었던 메타 분석에서 가장 낮은 수치 중 하나였을 것이다[11]. 환자와 의료진 모두 이차 항결핵제에 대한 경험이 없었던 치료 프로그램의 초창기에는 치료중단률이 훨씬 높았던 점을 감안할 때, 북한 내 치료 중단률이 이렇게 낮은 것은 매우 주목할 만하다. 북한 보건성과 유진벨 재단에서 환자와 의료진에게 끊임없이 교육하며 노력한 결과, 2012년부터 프로그램이 훨씬 안정화되었고 치료 중단률도 감소하였다.

낮은 치료 성과와 관련된 공변량(S1 문서와 S1 표)은 다제내성결핵 치료가 확대된다면 환자들이 시의적절하게 진단받고 효과적인 치료를 시작할 수 있어 완치율이 높아질 수 있다는 점을 시사한다. 이 환자들의 대다수는 양측성 병변과 공동 형성과 같이 병변이 매우 광범위하다. 이들 환자 중 17%는 폐가 손상됐고, 결핵이 악화된 징후를 보이고 있다. 거의 대부분의 환자들이 표준 DOTS 1부류 또는 2부류 처방에 속하는 일반 결핵 치료를 여러 차례 경험했는데, 이는 결핵균의 내성을 강화시키는 위험한 행위다[12].

효과적인 치료에 대한 접근성이 높아지면, 불규칙하거나 불충분한 치료로 초래된 이차 항결핵제에 대한 내성과 같은, 형편 없는 치료 성과의 위험요인이 해결될 것이다. 이 환자들의 XDR(광범위 내성 결핵) 비율은 6.7%, (MDR이면서 이차 항결핵제 주사액이나 플루오로퀴놀론 중 하나에 내성이 있는) Pre-XDR 비율은 19.1%(표2)이며, 이들 중 그 누구도 이차 항결핵제로 치료를 받은 적이 없다는 사실에 비추어볼 때, 이는 매우 놀라운 결과이다. 이는 비공식적으로 이차 항결핵제를 복용한 적이 있거나, 그런 환자로부터 전염이 되었다는 것을 의미한다. 보건성에서 이차 항결핵제를 철저히 관리감독 한다고 해서, 보건성 밖에서 이루어지는 이차 항결핵제에 대한 접근성을 완전히 제한할 수 있는 것은 아니다. 다른 나라에서도 다제내성결핵 환자들은 보통 젊고 의욕적이며, 검증되지 못한 경로로 소량의 이차 항결핵제라도 얻기 위해 그들이 쓸 수 있는 모든 자원을 다 활용한다. 안타깝게도, 이런 종류의 치료 방법은 일반적으로 불규칙적이고 비효과적이며, 때문에 종종 내성이 더욱 강한 결핵 균주를 야기하여 결국 가족과 이웃들에게까지 전염시킨다. Bedaquiline, delamanid, linezolid와 같은 신종 결핵 약은 내성이 매우 강한 결핵 균주의 치료 효과를 개선하기 위해 반드시 소개되어야 하지만, 만약 환자들이 치료에 대한 접근성을 갖지 못한다면 이러한 노력은 헛수고에 불과할 것이다.

**북한 내 다제내성결핵 치료 확대**

유진벨 재단 다제내성결핵 프로그램의 우수한 환자 치료결과는 북한 내 다제내성결핵의 진단 및 치료에 대한 재정지원이 왜 그렇게 소규모로 이루어지는지 의문을 제기한다. 환자들의 등록률은 꾸준히 증가하여 2008년 이후 현재까지 총 4,000명, 2015년 한 해에만 1,000명이 넘는 환자들이 등록했다(그림 2). 수천 명의 목숨을 구했다는 것에 의심의 여지가 없다. 그러나, 역학적 관점에서 이 프로그램을 보면, 이 수치는 관용구처럼 '코끼리에게 비스킷 주기' 수준이다. 아직도 북한에는 유진벨 재단이 전혀 지원하지 않는 도(道)도 있으며, 일부 지역에서는 요양소 1-2곳이 한 도(道)의 전 지역을 담당하고 있다.

**그림 2. 유진벨 등록 환자 누계 (2008-2015)**


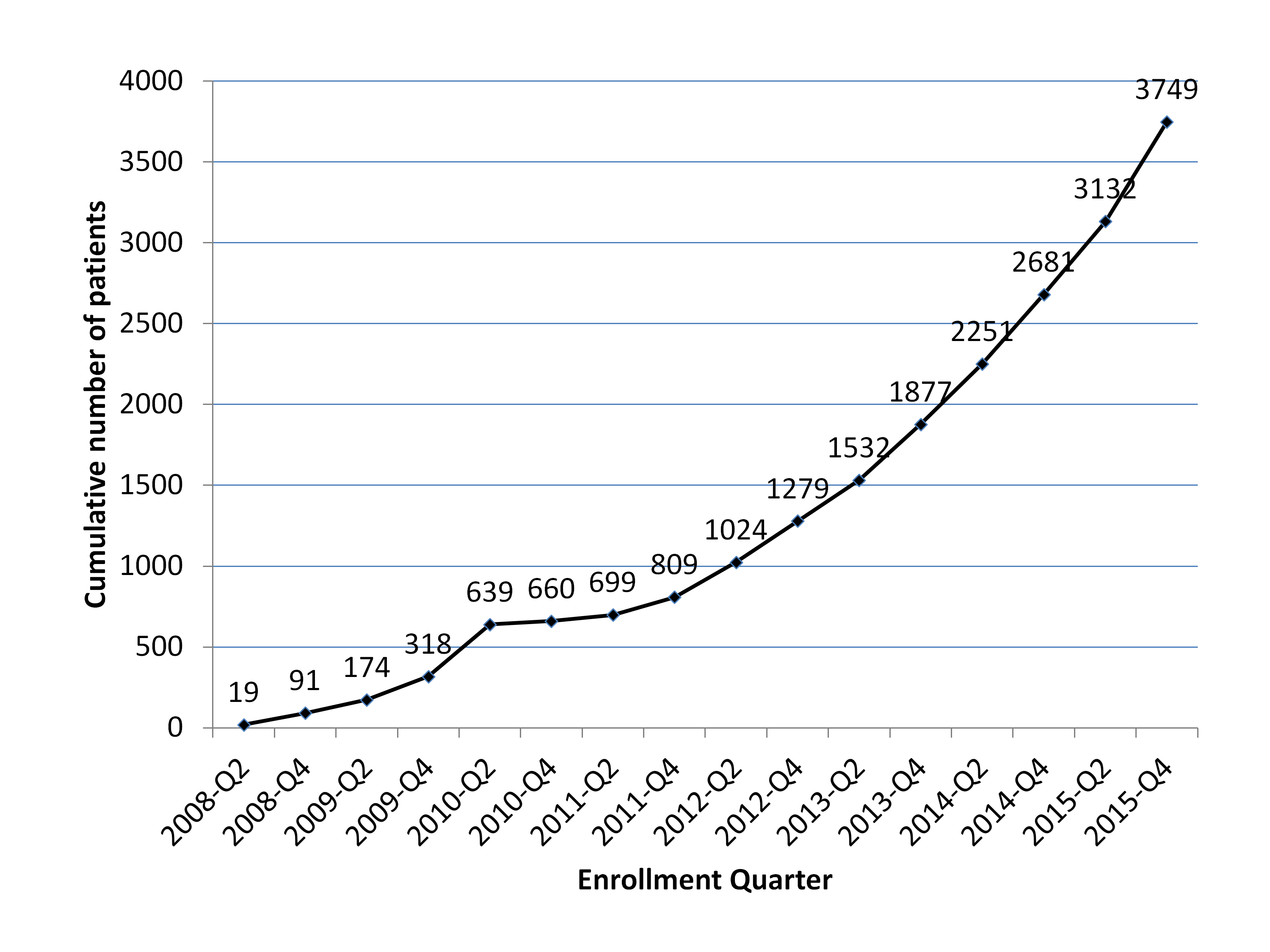


유진벨 재단 외에, 다제내성결핵의 진단 및 치료를 위한 국제사회의 지원 규모는 놀랄 만큼 작다. 2010년부터 북한 내 결핵 퇴치를 위해 가장 많이 지원해온 곳은 세계기금이다. 2015년 세계기금의 컨셉노트(Concept Note)에 따르면, 세계기금은 2016년에는 300명, 2017년 400명, 2018년 450명 다제내성결핵 환자를 치료할 계획이다[13]. 세계보건기구가 북한에서 한 해 3,800명의 다제내성결핵 환자가 새로 발병한다고 예측한 점을 감안하면, 이 수치는 역학적으로 의미가 없다[1].

다제내성결핵 진단 및 치료 비용이 지속적으로 낮아지는 점을 감안할 때, 다제내성결핵에 대한 재정지원을 이렇게 소규모로 한다는 결정은 매우 놀랍다. 현재 사용되고 있는 세계보건기구 진료치짐에 따르면, 재치료 환자(표준 DOTS 2부류), 활동성 폐결핵으로 진단 받은 약제내성결핵환자와 접촉할 가능성이 많은 자, 일차 항결핵제에 반응을 보이지 않는 환자(표준 DOTS 1부류에 실패자)에게 우선적으로 약제감수성 검사를 제공해야 한다[14]. 북한에서 이렇게 하려면, 한 해 약 15,000개의 Xpert^®^ MTB/RIF 카트리지가 필요하며, 연간 150,000달러의 비용이 소요될 것이다[15]. 유진벨 재단의 환자수가 증가하고 있는 현실을 감안하면, 다제내성결핵 치료를 확대하기 전에 북한전역의 악제내성률 조사를 먼저 시행해야한다는 주장은 무의미하다. 수 천명의 다제내성결핵 환자를 진단하는 데에는 많은 노력이 들지 않는다.

치료 확대에 따른 비용이 얼마이든 간에, 결핵의 유행을 방치함로써 발생하는 인명 손실과 그로 인한 비용과는 비교할 수 없이 작다. 효과적인 치료법에 대한 접근성이 확대되지 않는다면, 공기로 전염되는 다제내성결핵은 북한에서 계속해서 증가할 것이다. 최근 세계보건기구는 북한을 다제내성결핵으로 인한 질병부담이 높은 30개국의 하나로 지정하였지만, 국제사회의 대북지원은 거의 대부분 약제 감수성이 있는 일반 결핵에만 집중되어 있다. 유진벨 재단의 다제내성결핵 프로그램은 남한을 포함한 세계의 다른 국가들보다 치료 성적이 우수하다. 다제내성결핵 치료가 필요한 많은 북한 환자들을 위해 하루라도 빨리 프로그램이 확대되어야 한다.

**감사의 말**

다제내성결핵 환자를 돌보는 데 힘쓰는 북한의 많은 의사, 간호사를 비롯한 의료진과 보건성 관계자분들께 감사를 드립니다.

**데이터 이용가능성**

본 연구의 결과에 관련된 모든 데이터는 하버드 대학교 Harvard Dataverse(<http://dx.doi.org/10.7910/DVN/NKW22I>)에서 이용가능하다.

**윤리 심의**

이 연구는 파트너스 헬스케어 연구윤리위원회(Partners HealthCare Human Research Committee)의 연구 심의를 승인을 받았다. 이 연구는 일상적인 진료과정 중 과거에 수집된 자료를 바탕으로 한 후향적 연구이기 때문에, 승인된 연구계획서에서 사전동의서의 취득 의무가 필요성이 면제되었다.

**참고문헌**

^1^ World Health Organization. *Global tuberculosis control 2015* (WHO/HTM/TB/2014.22). Geneva: World Health Organization; 2015.

^2^ Seung KJ, Linton SW. The growing problem of multidrug-resistant tuberculosis in North Korea. *PLoS Med.* 2013; 10(7): e1001486.

^3^ Ahuja SD, Ashkin D, Avendano M, Banerjee R, Bauer M, Bayona JN, et al; Collaborative Group for Meta-Analysis of Individual Patient Data in MDR-TB. Multidrug resistant pulmonary tuberculosis treatment regimens and patient outcomes: an individual patient data meta-analysis of 9,153 patients. *PLoS Med*. 2012; 9(8): e1001300.

^4^ Mukherjee JS, Rich ML, Socci AR, Joseph JK, Virú FA, Shin SS, et al. Programmes and principles in treatment of multidrug-resistant tuberculosis. *Lancet*. 2004; 363(9407): 474-81.

^5^ The Global Fund [Internet]. Korea (Democratic Peoples Republic) [cited 21 Nov 2015]. Available from: <http://www.theglobalfund.org/en/portfolio/country/?loc=PRK>

^6^ World Health Organization. *Management of MDR-TB: A field guide* (WHO/HTM/TB/2008.402). Geneva: World Health Organization; 2009.

^7^ World Health Organization. *Guidelines for the programmatic management of drug-resistant tuberculosis: 2011 update* (WHO/HTM/TB/2011.6). Geneva: World Health Organization; 2011.

^8^ Pardini M, Varaine F, Iona E, Arzumanian E, Checchi F, Oggioni MR, et al. Cetyl-pyridinium chloride is useful for isolation of *Mycobacterium tuberculosis* from sputa subjected to long-term storage. *J Clin Micro.* 2005; 43(1): 442-444.

^9^ Kim DH, Kim HJ, Park SK, Kong SJ, Kim YS, Kim TH, et al. Treatment outcomes and survival based on drug resistance patterns in multidrug-resistant tuberculosis. *Am J Respir Crit Care Med.* 2010; 182: 113–119.

^10^ Jeon DS, Shin DO, Park SK, Seo JE, Seo HS, Cho YS, et al. Treatment outcome and mortality among patients with multidrug-resistant tuberculosis in tuberculosis hospitals of the public sector*. J Korean Med Sci*. 2011; 26: 33-41.

^11^ Toczek A, Cox H, du Cros P, Cooke G, Ford N. Strategies for reducing treatment default in drug-resistant tuberculosis: systematic review and meta-analysis. *Int J Tuberc Lung Dis.* 2012; 17(3): 299–307.

^12^ Farmer P. Managerial successes, clinical failures. *Int J Tuberc Lung Dis.* 1999; 3(5): 365-7.

^13^ The Global Fund [Internet]. Concept Note (2015 – Tuberculosis) [cited 21 Nov 2015]. Available from: http://www.theglobalfund.org/ProgramDocuments/PRK/ConceptNotes/2015/PRK-T_ConceptNote_0_en/

^14^ World Health Organization. *Companion handbook to the WHO guidelines for the programmatic management of drug-resistant tuberculosis* (WHO/HTM/TB/2014.11). Geneva: World Health Organization; 2014.

^15^ Foundation for Innovative New Diagnostics [Internet]. Price for Xpert® MTB/RIF and FIND country list [cited 2015 Aug 7]. Available from: http://www.finddiagnostics.org/about/what_we_do/successes/find-negotiated-prices/xpert_mtb_rif.html
